# Deep Learning Based Tongue Prickles Detection in Traditional Chinese Medicine

**DOI:** 10.1155/2022/5899975

**Published:** 2022-09-22

**Authors:** Xinzhou Wang, Siyan Luo, Guihua Tian, Xiangrong Rao, Bin He, Fuchun Sun

**Affiliations:** ^1^College of Electronic and Information Engineering, Tongji University, Shanghai 200092, China; ^2^Department of Computer Science and Technology, Tsinghua University, Beijing 100084, China; ^3^Guang'Anmen Hospital, China Academy of Chinese Medical Sciences, Beijing 100053, China; ^4^Beijing University of Chinese Medicine, Beijing 100029, China; ^5^Dongzhimen Hospital, Beijing University of Chinese Medicine, Beijing 100700, China

## Abstract

Tongue diagnosis is a convenient and noninvasive clinical practice of traditional Chinese medicine (TCM), having existed for thousands of years. Prickle, as an essential indicator in TCM, appears as a large number of red thorns protruding from the tongue. The term “prickly tongue” has been used to describe the flow of qi and blood in TCM and assess the conditions of disease as well as the health status of subhealthy people. Different location and density of prickles indicate different symptoms. As proved by modern medical research, the prickles originate in the fungiform papillae, which are enlarged and protrude to form spikes like awn. Prickle recognition, however, is subjective, burdensome, and susceptible to external factors. To solve this issue, an end-to-end prickle detection workflow based on deep learning is proposed. First, raw tongue images are fed into the Swin Transformer to remove interference information. Then, segmented tongues are partitioned into four areas: root, center, tip, and margin. We manually labeled the prickles on 224 tongue images with the assistance of an OpenCV spot detector. After training on the labeled dataset, the super-resolutionfaster-RCNN extracts advanced tongue features and predicts the bounding box of each single prickle. We show the synergy of deep learning and TCM by achieving a 92.42% recall, which is 2.52% higher than the previous work. This work provides a quantitative perspective for symptoms and disease diagnosis according to tongue characteristics. Furthermore, it is convenient to transfer this portable model to detect petechiae or tooth-marks on tongue images.

## 1. Introduction

**Figure 1:**
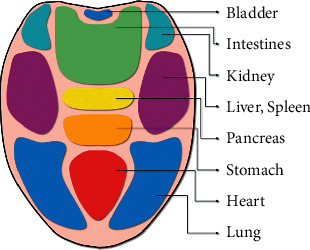
Tongue reflexology chart. Different areas on the tongue reflect the state of different organs.

Based on the clinical practice of doctors, traditional Chinese medicine has been developed for thousands of years and has achieved brilliant results both in the past and in modern times. Tongue diagnosis, as a role of vital importance in TCM clinical diagnosis, is a convenient and noninvasive method based on the health status information carried by the appearance of the tongue [1]. The chromatic features and morphological characteristics of the tongue, the number of prickles and the form of tongue coating reveal the pathological changes of internal organs, as shown in Figure 1 [2, 3]. There have been reports that prickles are associated with tumors, kidney disease, gastric disease, and new crowns [4]. However, traditional tongue diagnosis is an empirical procedure that relies heavily on the personal experience and subjective judgment of TCM doctors. With the assistance of artificial intelligence (AI), tongue diagnosis will be objective and people without medical knowledge can give themselves a preliminary diagnosis of a health condition. In recent years, much effort has been spent on AI-based tongue diagnosis, especially in the field of tongue color recognition [5, 6], tongue shape analysis [7], cracks segmentation [8], thickness, and moisture of tongue coating classification [9, 10].

Prickle, also called red-pointe, appears as a large number of red thorns protruding from the tongue. It indicates blood heat or excess heat in the internal organs. The color and number of the prickles can help estimate the flow of qi and blood. As proved by the study, the prickles originate in the fungiform papillae, which are enlarged and protrude to form spikes like awn [11]. Shang et al. further analyzed the association of prickles with the gastric sinus [12]. On the one hand, prickles mean increased blood flow and thus congestion. On the other hand, prickles represent thermal burns to blood vessels, resulting in blood spillage and mucosal erythema. The automatic detection of prickles can not only release the burden of doctors but also enable patients without medical knowledge to give themselves a brief examination.

Though AI-based tongue diagnosis has attracted a lot of attention, there is little literature on prickle detection due to its difficulty. In most tongue images, each prickle only occupies a few pixels and has little difference in tongue color under natural light. Moreover, the similarity between prickles and petechiae in both morphological and chromatic characteristics makes it a challenging task to distinguish them. Xu et al. were the first to introduce template feature matching to detect the prickles and petechiae, and then distinguished them based on RGB value range, gray average, and the position of the detected object [13]. Zhang employed the fuzzy *C*-means color cluster and noise reduction methods to detect prickles in the tongue edge image. Wang et al. used multistep threshold spot detection to detect prickles and petechiae. After extracting the features of spots (including prickles and petechiae), support vector machines and *k*-means were introduced to distinguish prickles and petechiae [11]. Wang et al. proposed a prickle detection method based on an auxiliary light source and a LOG operator edge detection method [14]. The last two works are most similar to our work, for they provided a quantitative description of prickles.

By eliminating background areas such as the face and lips, the tongue region segmentation can enhance the performance of downstream tasks, including prickle detection. Practice has proved that the neural network is very effective in the task of tongue segmentation. Zhang et al. introduced a DCNN-based tongue segmentation algorithm [15]. Wang et al. designed a coarse-to-fine segmentor based on RsNet and FsNet [16]. Jiangproposed an HSV enhanced CNN to segment the tongue region [17]. Zhang et al. combined superpixel with CNN to increase decoding performance [18].

Though previous researchers put much efforts into prickles and petechiae detection, the existing methods all rely heavily on manual parameter tuning. This not only adds to the burden of researchers but also causes the model to overfit to specific circumstances and equipment. Moreover, there is no end-to-end prickle detection method, which could provide a quantitative description of prickles without manually segmenting the tongue raw images. Finally, most methods only took gray values of the exact pixels into consideration and lose the color information and the context information around the prickles.

**Figure 2:**
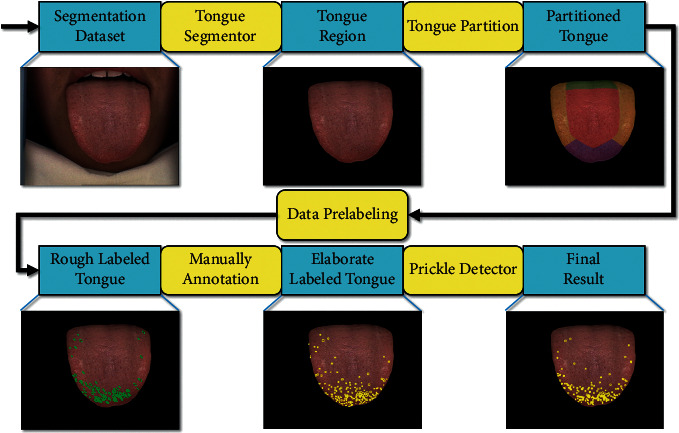
Prickle detection workflow. Blue rectangles represent images, and yellow rectangles represent image processing. First, in tongue segmentor, we introduced Swin Transformer, a state-of-the-art computer vision segmentation neural network, to mat the tongue region out of the raw picture. Second, in tongue partition, the tongue is partitioned into four areas: root, margin, tip, and center. Third, in data prelabeling, a spot detector is applied based on tongue areas. Fourth, elaborate manual annotation is conducted with the help of TCM doctors. Fifth, to fully embody the advantages of the neural network, a super-resolutionfaster-RCNN based detector is deployed to detect the prickles from a matted tongue image.

The method proposed in this paper solved the question mentioned above from a completely new perspective: Deep Learning. We designed an end-to-end workflow to detect prickle automatically. The entire workflow and intermediate results are depicted in Figure 2.

## 2. Dataset and Methods

### 2.1. Dataset Collection

**Figure 3:**
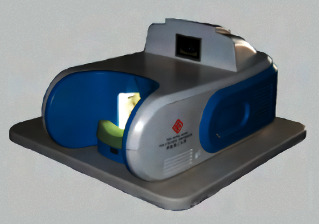
The tongue image acquisition device. The device is designed to obtain tongue images in uniform illumination and facial poses.

In this paper, the tongue images and segmentation annotations come from the bio-HIT tongue image dataset [19] (https://github.com/BioHit/TongeImageDataset). The tongue images dataset contains 300 RGB images with 576 × 768 pixels, and the images are obtained by the tongue image acquisition device shown in Figure 3. The device is designed as a semiclosed black box with a camera and illuminated on each side of the camera. The daylight illuminant D50, recommended by CIE (Commission Internationale de lEclairage) [20], was utilized as daylight illumination. According to the guidelines provided by CIE, the angle between the incident and outgoing rays is 45°. We elaborately screened out images with poor quality and got 224 images to train the model. The device has a closed image acquisition environment with an independent stable light source and a head restraint to ensure all the images are sampled to one standard. In addition, the image registration and calibration is not necessary either. Four volunteers in HIT elaborately annotated the image segmentation labels and the best one was chosen [19]. All the images were standardized to meet the standard normal distribution.

### 2.2. Tongue Segmentation

**Figure 4:**
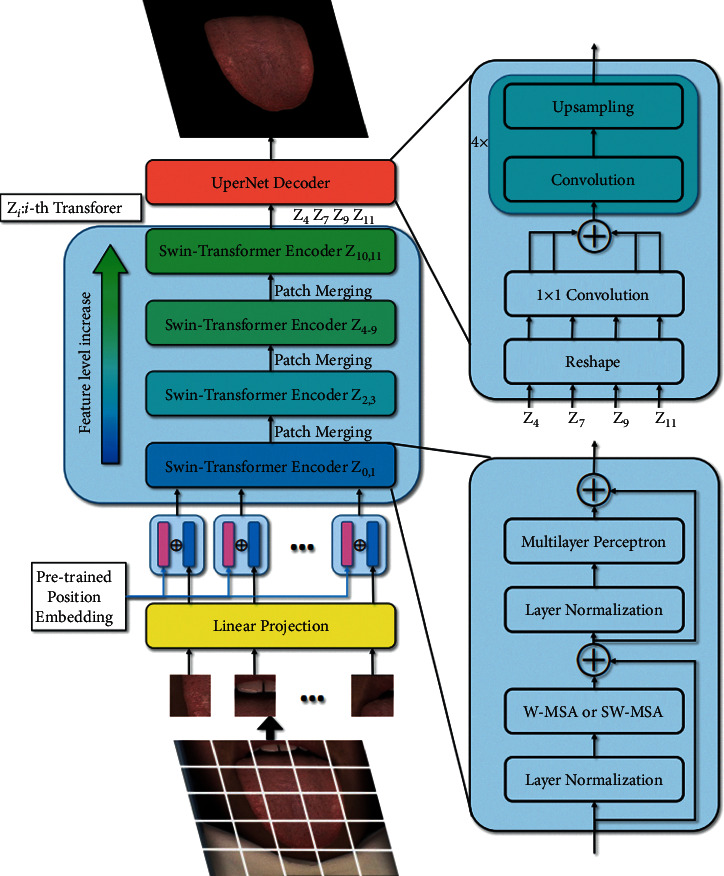
Architecture of tongue segmentor. The tongue image is divided into several patches and then added with position embeddings to retain spatial information. The encoder consists of cascaded Swin Transformers, while UperNet decoder is applied to aggregate multilevel features from encoder.

**Figure 5:**
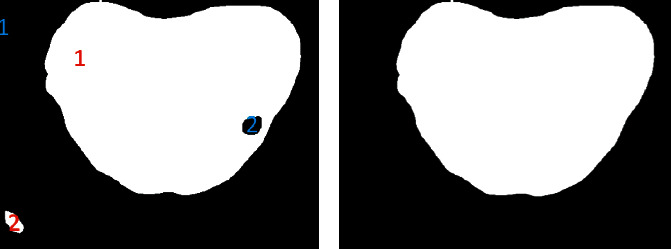
Mask morphology processing. The connected component with the largest area in black or white will be marked as “1” and other connected components will be eliminated.

The AI-assisted tongue diagnosis is based on the information obtained from the tongue image. When concentrated on the tongue, irrelevant elements, including the lips, cheek, and chin, distract I neural network. With interference eliminated and the tongue matted, the contour line of the tongue becomes apparent and the performance of feature extraction is guaranteed. Therefore, it is necessary to segment the tongue before the next step, and we introduced the Swin Transformer [21] as the segmentor. The core concept of the Swin Transformer is self-attention, as shown in equations:

(1) $$\begin{array}{c} \begin{array}{c} K=XW^{K},\\ V=XW^{V}, \end{array} \end{array}$$

(2) $$\begin{array}{c} , \end{array}$$

where the *Q* is the query vector, the *K* is the key vector, and the *V* is the value vector. *W*^*Q*^, *W*^*K*^, *W*^*V*^ are all weight matrices. With the self-attention mechanism, the network can perceive global semantic information. The entire architecture of Swin Transformer is shown in Figure 4, where *Z*_0_, *Z*_2_, *Z*_4_, *Z*_6_, *Z*_8_, and *Z*_10_ used multihead self-attention with regular windowing (W-MSA) and the others used multihead self-attention with shifted windowing (SW-MSA). Considering the fact that the dataset only contains 224 images, which is insufficient for training a network from scratch, we adopted a paradigmatic strategy in computer vision: pretraining and fine-tuning with data augmentation. Microsoft has trained the model with over 20,000 images on the ADE20K dataset [22], and we fine-tuned the model on the tongue segmentation dataset. The data augmentation pipeline includes flipping, cropping, and photometric distortion. Photometric distortion applies the following transformations with a probability of 0.5: random brightness, random contrast, color space converting, random saturation, random hue, and randomly swapping channels. With data augmentation, the model will be more robust when the illumination or sampling device varies. Though the neural network is able to classify each pixel as tongue or background, there is no guarantee that the segmented tongue region has structural integrity. To address this issue, we analyzed the connected components of each mask, filled the blank areas in the tongue and eliminated the outliers using two-pass connected component analysis [23]. The algorithm is shown in Algorithm 1 and the result is shown in Figure 5.

### 2.3. Prickles Annotation

Usually, hundreds of prickles appear on the tongue in groups. Therefore, it will be a challenging task if we manually annotate the whole dataset. In this paper, we employed partition spots detection [11] with LAB chromatic aberration filtering to give a primitive annotation of the prickles.

The LAB color space is based on the human eye's perception of color and can represent all colors the human eye can perceive. “L” represents lightness, “A” represents red-green difference, and “B” represents blue-yellow difference. The spot detection algorithm works on gray images and the color information is lost. Therefore, we filtered the spots by the chromatic aberration between the spots and the manually picked prickles. Given an RGB value, an approximate LAB chromatic aberration can be calculated as follows:

(3) $$\begin{array}{c} \tilde{r}=C_{1,R}+\frac{C_{2,R}}{2},\\ \Delta R=C_{1,R}-C_{2,R},\\ \Delta G=C_{1,G}-C_{2,G},\\ \Delta B=C_{1,B}-C_{2,B},\\ \text{Chromatic Aberration}=\sqrt{\left(2+\frac{\tilde{r}}{256}\right)\times \Delta R^{2}+4\times \Delta G^{2}+\left(2+\frac{\left(255-\tilde{r}\right)}{256}\right)\times \Delta B^{2}}. \end{array}$$

The tongue is partitioned before detection so that we can elaborately set different detection parameters for different areas. The tongue coating is distributed on the tongue surface, which is usually slightly thicker in the center or root of the tongue, and the prickles covered by the coating have different characteristics from the prickles on the margin and tip. In addition, the cracks on the root and center of the tongue tend to be detected mistakenly by the spot detection algorithm. To solve this problem, we divided the tongue into four areas: root, margin, tip, and center before preliminary annotation. Then we set the threshold of chromatic aberration, area, circularity, and convexity tighter in the root and center than in other areas. This setting avoids the misdetection of cracks while maintaining the detection rate.

**Figure 6:**
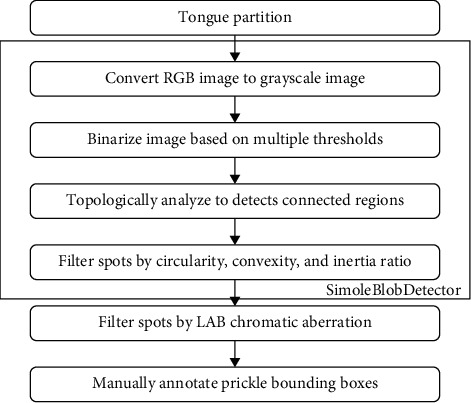
Prickle bounding boxes annotation workflow. Prickle bounding boxes are labeled automatically and then manually adjusted by TCM doctors.

**Figure 7:**
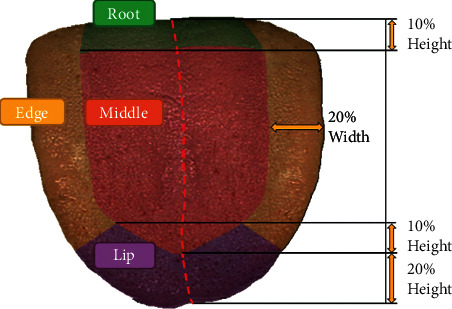
Tongue partition. The tongue is divided into four areas automatically based on the midline and the detection parameters vary with areas adaptively.

The algorithm of annotation is shown in Figure 6. First, the parallel-line method is introduced to build a reference line for tongue partition [14]. Second, the tongue is divided into four areas by the relative thickness compared to the overall scale of the tongue. The result is depicted in Figure 7. Third, a simple blob detector based on OpenCV (Open Source Computer Vision Library) is deployed with different parameters in different tongue regions. Fourth, the detected spots are filtered by LAB chromatic aberration. Finally, a professional TCM doctor revised the roughly annotated bounding boxes with the MIT Labelme annotation tool (https://labelme.csail.mit.edu) and two other TCM doctors checked the result under the same diagnostic criteria on the same monitor [24, 25].

### 2.4. Prickle Detection

**Figure 8:**
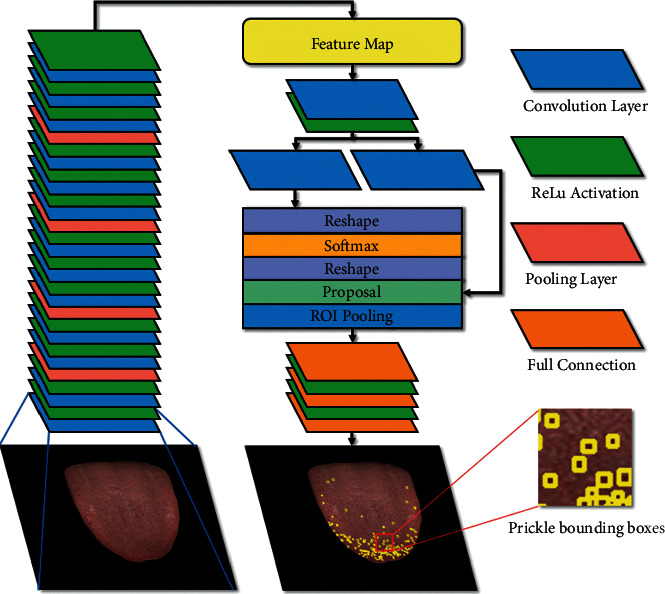
Architecture of prickle detector. CNN encoder extract features from images. Region proposal networks generate region proposals and bounding box regressor modify anchors to predict precise prickle bounding boxes.

In this paper, we take the Faster-RCNN as the prickle detector. In 2016, Ross B. Girshick proposed a new object detection neural network called Faster-RCNN, which is depicted in Figure 8. The Faster-RCNN first uses a set of basic convolution layers, ReLU function, and pooling layers to extract an image feature map, which is subsequently shared by region proposal networks (RPN) and fully connected layers. The RPN network is designed to generate region proposals with the softmax layer to determine whether the anchors are positive or negative, and then it employs bounding box regression to correct the anchors to obtain accurate proposals. The roi-pooling layer collects the input feature maps and proposals, extracts proposal feature maps after synthesizing the information, and sends them to the subsequent fully connected layer to determine the target category [26].

**Figure 9:**
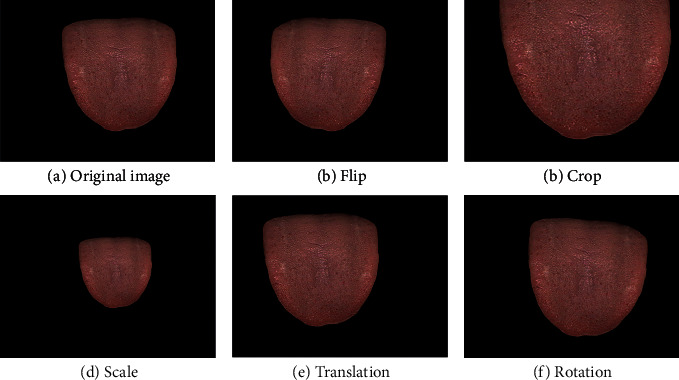
Data augmentation. Super-resolution, flip, crop, scale, translation, and rotation are conducted to increase dataset size and improve network robustness without collecting new data. (a) Original image (b) flip (c) crop (d) scale (e) translation and (f) rotation.

There are two obstacles that need to be overcome before training the Faster-RCNN. First, insufficient data makes the model hard to train. Therefore, we introduced data augmentation, including flip, crop, scale, translation, and rotation, as shown in Figure 9. In addition, we followed the paradigm of computer vision workflow: we pretrained the model on a general detection dataset and used transfer learning to fine-tune the model on the prickle detection dataset. Second, the Faster-RCNN is designed for the detection of the target on a normal scale. When the target is smaller than 32 × 32 pixels, the performance of the model will decrease sharply. To address this issue, we used 4× bilinear interpolation for upsampling to improve the model performance. Since the neural network aims to build an end-to-end prickle detection model and it is proven that the color calibration and irrelevant noise filtering may cause a degradation in model performance [27], image registration and filtering are not employed.

### 2.5. Evaluation Metrics

**Figure 10:**
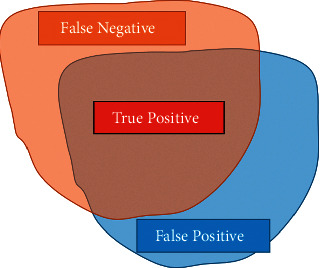
Types of the predicted segmentation. The orange area represents the ground truth, and the blue represents the predicted segmentation.

The segmentation tasks in computer vision field could be considered as a pixel-wise classification, and there are four types of the results: true positive (TP), false positive (FP), true negative (TN), and false negative (FN), as shown in Figure 10. The standard evaluation metrics of segmentation task is intersection over union (IoU), defined as follows:

(4) $$\begin{array}{c} \begin{array}{c} \text{IoU}=\frac{\text{TP}}{\left(\text{FP}+\text{FN}+\text{TP}\right)} \end{array}, \end{array}$$

where TP, FP, and FN are about pixel-wise classification results. We also employed precision and accuracy as metrics to fully demonstrate the performance of the proposed method, which are defined as follows:

(5) $$\begin{array}{c} \text{Accuracy}=\frac{\left(\text{TP}+\text{TN}\right)}{\left(\text{TP}+\text{TN}+\text{FP}+\text{FN}\right)}\text{,}\\ \text{Precision}=\frac{\text{TP}}{\text{TP}+\text{FP}}. \end{array}$$

A prickle is classified as detected correctly when the bounding box has IoU over the threshold.

The metrics for prickles detection are precision defined above and recall defined as follows:

(6) $$\begin{array}{c} \text{Recall}=\frac{\text{TP}}{\left(\text{TP}+\text{FN}\right)}\text{.} \end{array}$$

Where FP is the number of misdetected prickles, TP and FN is the number of manually annotated prickles that were detected and undetected, respectively.

## 3. Experiments and Results

### 3.1. Experiment Setting

In our work, the models were trained and tested based on the Python deep-learning framework PyTorch (https://pytorch.org) and the computing platform is a Linux server with Intel Xeon (R) E5-2620 CPU, 4 NVIDIA RTX2080Ti GPUs, and 128 GB memory. The total training time is about 2 hours. The parameters of segmentor and detector in the training stage are shown in Table 1.

### 3.2. Result of Tongue Segmentation

**Figure 11:**
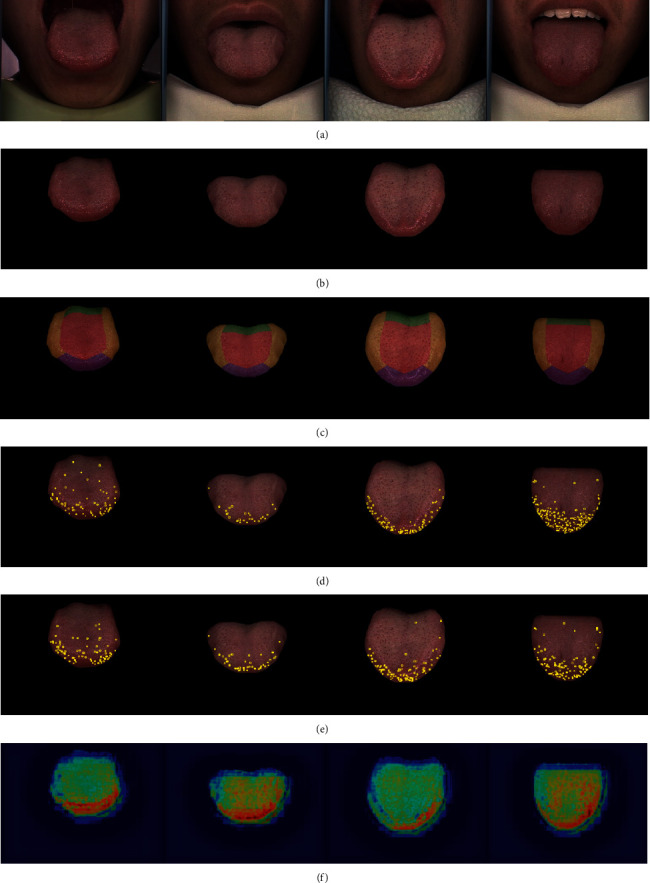
Prickle detection result. (a) Original images (b) segmentation result (c) partitioned tongues (d) prickle labels (e) prickle detection result and (f) neural network attention map. The intermediate result of the whole workflow is shown (zoom in for better viewing).

The Swin Transformer is a flexible neural network, which means it requires more training compared to the conventional CNN. To address this issue, we introduced the pretraining model provided by Microsoft (https://github.com/SwinTransformer/Swin-Transformer-Semantic-Segmentation). Microsoft has trained the model with over 20,000 images on ADE20K dataset [16] and we fine-tuned the model on our tongue segmentation dataset. The training set and test set contained 178 and 46 annotated images, respectively. The splitting of the dataset took a cross-validation strategy. After we got the predicted segmentation, we filled the blank areas in the tongue and eliminated the outliers. The result is shown in Table 2 and Figure11(b).

The excellent performance of the Swin Transformer makes the segmentation results basically the same as manual segmentation. Compared to the conventional machine vision segmentation methods, neural network is an end-to-end progress without the requirement of manual tuning parameters. It is also more robust when the illumination or sampling device varies and the result proves the superiority of the transformer architecture in tongue segmentation.

### 3.3. Prickle Labeling

We employed the simple blob detector with LAB chromatic aberration filtering to detect the prickles coarsely. The simple lob detector is a multistep threshold spot detection method for processing gray images. We took the parameters of the simple blob detector in [11] as the initial value and introduced the grid search to find the optimal parameters for each area. The searching step length is set to 10% of the initial value and the searching range is set from 50% to 150% of the initial value. The parameters of the partitioned simple blob detector are shown in Table 3.

**Figure 12:**
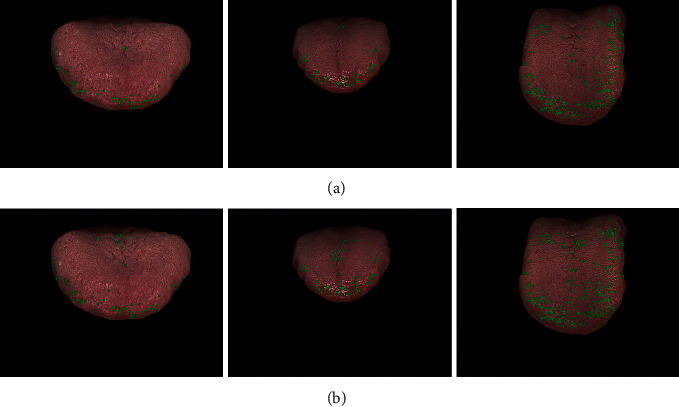
Automatic annotation results with and without partitioning. (a) Annotation with partitioning. (b) Annotation without partitioning. With annotation parameters differing between areas, the misdetections of center cracks are reduced.

Petechiae are usually found on the center and roots of the tongue. To reduce the probability of misdetection, the constrains of spots in those areas are more stringent. In addition, to take full advantage of the color information, we sampled the RGB value of prickles in different tongues and areas and filtered the detected spots by the LAB chromatic aberration. The automatic annotation result with and without partitioning is shown in Figure 12 to give a clear demonstration of how the partitioning helps the prickle labeling. After automatic annotation, three well-trained TCM doctors manually revised the labels. The annotation is shown in the Figure 11(d).

### 3.4. Result of Prickle Detection

Object detection is a challenging task requiring a lot of training. We downloaded the pretrained model provided by CUHK and SenseTime (https://github.com/open-mmlab) and fine-tuned it on the prickle detection dataset. The training set and test set contained 178 and 46 annotated images, respectively. The splitting of the dataset took a cross-validation strategy. Compared to the original Faster-RCNN, we employed a 4× bilinear interpolation for upsampling and modified the anchor size to match the prickle detection. To demonstrate the superiority of our modified Faster-RCNN, we took the vanilla Faster-RCNN and other detection algorithms for comparison. The predicted result of the entire dataset is shown in Table 4.

The recall of our method outperformed the existing methods without fine-tuning the parameters manually. We did not choose YOLO because it is a one-stage detection algorithm and the resolution is fixed, which limits the performance of detecting tiny targets. It is worth mentioning that we tried other learning-based algorithms, including DCNv2 [29] and SSD [30], but they were unable to predict any bounding box. As shown in Figure 11(e), the detected prickle patterns are similar to the annotation while it tends to detect prickles on the tip. To illustrate the principle of our neural network, we provided an attention heat map of the detector, as shown in Figure 11(f). The neural network had more attention on the edge of the tongue, where the prickles are most likely to appear. This attention is learned by the neural network automatically and has the potential to provide TCM doctors an intuition to distinguish a tongue with prickles. In addition, we fed the raw images into the detector as an ablation experiment to validate the necessity of segmentation, and the result is shown in the 3rd line of Table 4.

## 4. Discussion

Prickle, as an essential syndrome feature of TCM, can be used to assist in the diagnosis and treatment of subhealthy people. But the ambiguity of prickle recognition and the subjective preferences of doctors limit its further application. Combined with modern AI technology, we proposed an end-to-end computer vision-based prickle detection workflow, which makes prickle detection more precise and objective. This workflow is divided into three main steps. Firstly, Swin Transformer, a state-of-the-art semantic segmentation neural network, was employed to segment the tongue region out of a raw image. The segmented tongues were partitioned into four areas: root, center, tip, and margin to help the parameters setting of follow-up spot detection and prickle detection. Secondly, we manually labeled the prickles on 224 tongue images with the help of a spot detector and fed the result into the Faster-RCNN. Finally, the neural network extracted the features of images at both a texture level and morphological level to detect the prickles on the tongues. The precision of our segmentation is 99.47%, and the recall of our detection is 92.42%, which outperformed the existing methods. The result illustrates that the utilization of transfer learning made it possible to train neural networks on a limited number of images. Compared to previously published studies, it gets rid of the trivial parameters tuning procedure and releases the burden on researchers. Meanwhile, the workflow proposed is more portable, which means you can transfer the model to arbitrary tongue characteristics or image acquisition equipment.

In the context of artificial intelligence and big data, the informatization of TCM diagnosis is an area that urgently needs in-depth research. Our work took full advantage of the deep learning algorithm to implement an intelligent recognition of prickles and provided the possibility of establishing the quantitative association between the tongue image and clinical symptoms. In addition, incorporating a prickle detection model into the smartphone will allow people without medical knowledge to give themselves a simple health status assessment, and a quantitative and objective tongue diagnosis will also benefit the integration of TCM and modern Western medicine. The precise segmentation and feature recognition of the tonguecan also be used for throat swab robot perception.

Though our method is state-of-the-art, there are some aspects for further research. Firstly, the model was trained on the images sampled by a standard acquisition device, which limits its generalization and robustness. A larger dataset consisting of images from different devices would help the model to establish a greater degree of accuracy in this matter. Secondly, our model provides a paradigm for tongue feature detection. With petechiae, cracks, tooth-marks, and other TCM features of the tongues labeled, the model has the potential to achieve an acceptable result. Thirdly, most existing learning-based tongue feature detection methods aim to find bounding boxes. It is possible to use a segmentor to classify every single pixel in the image into a kind of TCM tongue feature.

## Figures and Tables

**Algorithm 1:**
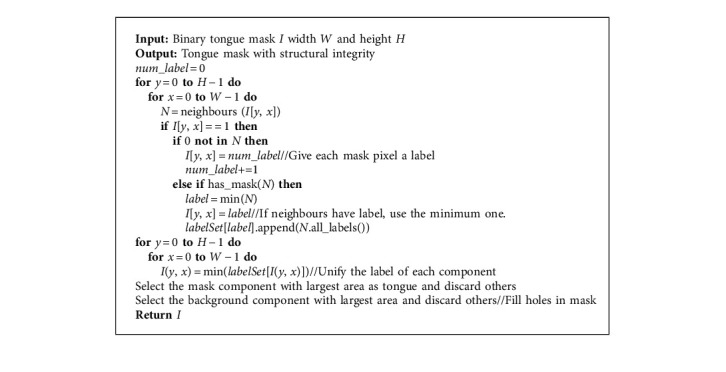
Mask morphology processing.

**Table 1:** Parameters for training Swin Transformer.

Hyper-parameter	Segmentor	Detector
Epoch	20	100
Batch size	4	4
Optimizer	AdamW	SGD
Learning rate	6e-05	5e-2
Learning rate policy	Polynomial	Step
Weight decay	1e-2	1e-4
Loss function`	Cross entropy	Cross entropy

**Table 2:** The performance of segmentor and previous studies.

Method	IoU (%)	Precision (%)	Accuracy (%)
DCNN [8]	—	97.94	99.41
RsNet and FsNet [9]	—	97.85	99.04
HSV enhanced CNN [10]	—	94.70	97.88
Ours	**99.08**	**99.47**	**99.79**

**Table 3:** Parameters of the simple blob detector in different areas.

Parameter	Root and center	Tip	Margin
Min threshold	60	60	60
Max threshold	100	100	100
Threshold step	2	2	2
Min repeatability	8	4	4
Min area	4	2	2
Max area	25	40	40
Min circularity	0.8	0.4	0.4
Min convexity	0.8	0.4	0.4
Min inertia ratio	0.5	0.4	0.4
Max aberration	85	100	100

**Table 4:** Prickle detection results and comparison with previous studies.

Method	Recall				Accuracy
	Root & center (%)	Tip (%)	Margin (%)	Total (%)	Total (%)
LOG [7]	84.97	67.37	84.23	86.95	—
SVM [5]	—	—	—	89.90	—
YOLO [28]	73.28	67.68	65.48	67.95	60.44
Vanilla faster-RCNN [26]	69.43	66.48	61.22	65.58	72.13
Ours w/o segmentation	88.89	89.45	83.34	89.77	86.69
Ours	**98.62**	**89.92**	**94.59**	**92.42**	**88.46**

## Data Availability

The experimental data and code used to support the findings of this study will be available on https://github.com/zz7379/PrickleDetection or on contacting the authors with reasonable request (wangxinzhou@tongji.edu.cn).
